# Dynamics and stability of three-dimensional ferrofluid films in a magnetic field

**DOI:** 10.1007/s10665-017-9938-2

**Published:** 2017-09-15

**Authors:** Devin Conroy, Omar K. Matar

**Affiliations:** Department of Chemical Engineering, Imperial College London, South Kensington Campus, London, SW7 2AZ UK

**Keywords:** Fingering, Interface, Interfacial flows, Long-wave approximation, Lubrication theory, Magnetic fields, Numerical simulations, Stability, Thin films

## Abstract

We consider the interfacial dynamics of a thin, ferrofluid film flowing down an inclined substrate, under the action of a magnetic field, bounded above by an inviscid gas. The fluid is assumed to be weakly conducting, and its dynamics are governed by a coupled system of the steady Maxwell, Navier–Stokes, and continuity equations. The magnetization of the film is a function of the magnetic field, and is prescribed by a Langevin function. We make use of a long-wave reduction in order to solve for the dynamics of the pressure, velocity, and magnetic fields inside the film. The potential in the gas phase is solved by means of Fourier Transforms. Imposition of appropriate interfacial conditions allows for the construction of an evolution equation for the interfacial shape, via use of the kinematic condition, and the magnetic field. We study the three-dimensional evolution of the film to spanwise perturbations by solving the nonlinear equations numerically. The constant-volume configuration is considered, which corresponds to a slender drop flowing down an incline. A parametric study is then performed to understand the effect of the magnetic field on the stability and structure of the interface.

## Introduction

Thin film flows are of central importance to numerous industrial, biomedical, and daily-life applications [1, 2]. These include coating flow technology, surfactant replacement therapy, chemical reaction engineering, heat and mass transfers, and process-intensification applications. The flows can be driven by various surface and body forces such as gravitational, Marangoni (solutal and thermocapillary-driven), centrifugal, intermolecular, as well as electromagnetic ones. We focus in this paper on the dynamics of ferrofluids, which are colloidal-based fluids comprising nanosized magnetic particles, stabilzed by surfactants in a viscous liquid. The body forces in ferrofluids arise due to polarization from the presence of a magnetized material in a magnetic field [3]. Thus, these fluids can be manipulated via an externally imposed magnetic field [4–6], with applications in rotary-shaft seals [3], pharmaceuticals [7], self-assembly [8], and biomedical settings [9, 10].

Free surface flows involving ferrofluids have received some attention in the literature, starting with the linear stability analysis of Cowley and Rosensweig [11] who demonstrated the presence of the so-called *normal field instability* (NFI). This corresponds to the transition of a magnetic film of initially uniform thickness into an array of hexagonal, crest-like structures in the presence of a magnetic field oriented perpendicularly to the film. The NFI, which occurs beyond a critical value of the magnetic induction, has since been studied by Zelazo and Melcher [12] who showed that normal and tangential magnetic fields are destabilizing and stabilizing, respectively. More recently, M$\ddot{\mathrm{u}}$ller [13] showed that viscosity can delay the onset of NFI, and Gollwitzer et al. [14] found good agreement with the work of Cowley and Rosensweig [11] through a Langevin function for the relationship between magnetization and the magnetic field; a similar function will be used in the present paper.

For thin film flows, Joo [15] used the long-wave approximation to derive a nonlinear evolution equation for the ferrofluid film accounting for weak magnetic effects. Moulton and Pelesko [16] used a combination of lubrication theory and experiments to study ferrofluid film drainage. In these models, magnetic effects are accounted for through the inclusion of Maxwell’s stresses at the interface and in the bulk. The relation between the magnetic field and the magnetization in the case of ferrofluids is either linear [12] or, for sufficiently large magnetic fields, nonlinear [6]; in the former case, it is possible to rationalise ferrofluid flows by analogy with their electrified counterparts [17–21].

The instabilities exhibited by flows involving thin films and slender drops have been well studied [1, 2]. In situations where the film, or drop, flow down an incline, a front develops at the film/drop leading edge, which is unstable to spanwise perturbations, under certain conditions. Investigations of the front dynamics tend to fall into either the ‘constant-flux’ [22] or ‘constant-volume’ [23] cases. For the case of a constant-volume of fluid released on an inclined plane, a capillary ridge forms behind a contact line, and eventually becomes unstable to spanwise perturbations. Over time, finger-like structures form, and it was first shown by Huppert [24] that the shape of the patterns can be determined by the contact line dynamics. Also, the presence of the contact line creates a paradox when the no-slip boundary condition is used, leading to multivalued velocity fields [25, 26]. To overcome this issue, it is common to apply a slip condition, or include a precursor film ahead of the contact line.

The presence of a ferrofluid in the fingering instability problem will lead to a new effect that has not yet been explored. Due to the magnetic particles that are dispersed within the fluid, a Maxwell pressure is generated following the application of a magnetic field. It is possible to examine the effect of a magnetic field on the fingering instability of a thin film or slender drop using the long-wave approximation [1, 2] to couple the interfacial dynamics to the Maxwell stresses. Using this approach, the case of a highly conducting ferrofluid with a nonlinear magnetic susceptibility was investigated by Conroy and Matar [6] who found that the magnetic field has a destabilizing effect and led to a complex surface structure that increased in amplitude with increasing magnetic pressure; this work, however, was restricted to two-dimensional flows.

In this paper, we extend the model developed by Conroy and Matar [6], for the long-wave evolution of a ferrofluid film, to three dimensions. We solve the resultant equations numerically using pseudo-spectral methods, and investigate the spanwise instability of a falling drop down an inclined plane; the magnetic effects enter through a Maxwell pressure. We find that increasing the Maxwell pressure causes the front velocity and the amplitude of the ridge formed at the advancing contact line to increase. We also find magnetic effects to be destablizing leading to finger-formation, with their growth rate increasing with the Maxwell pressure. In addition, the fingers develop spike-like structures that continue to advance downstream from the initial point of deposition. The amplitude of these structures was also found to increase with magnetic field strength.

The rest of the paper is organized as follows. In Sect. 2, we formulate the three-dimensional model for a thin ferrofluid film in a magnetic field, and use long-wave theory to derive a single equation for the interface. In Sect. 3, we provide a discussion of the numerical solutions of this equation, focusing on the stability characteristics of the interface. Finally, in Sect. 4, we provide concluding remarks.

## Formulation

We consider the dynamics of a ferrofluid film with magnetization $\mathbf{M}$ on a solid, rigid, and impermeable plane, inclined at an angle $\theta$ to the horizontal. We use a rectangular coordinate system (*x*, *y*, *z*) to model the film flow in which the wall is located at $z=-z_w$. The interface, which has a constant surface tension $\gamma$, is defined by $z=S(x,y,t)$, and separates the film from the surrounding gas. Far away from the film, a magnetic field is applied in a direction normal to the inclined plane. The substrate beneath the film is assumed to be maintained at a constant magnetic field $\mathbf{H}_w$. Here, we consider the constant-volume configuration, corresponding to a drop placed on a substrate, with a thin precursor film used to relieve the stress singularity at the moving contact lines.

### Governing equations

The governing equations describing ferrofluid films were presented previously in the work of Conroy and Matar [6]; thus, we only provide the dimensionless form, suitably extended to three dimensions. We scale the vertical dimension with the initial film height, $z_w$, as $(z,S)=z_w({\check{z}},{\check{S}})$, the horizontal dimensions as $(x,y)=L({\check{x}},{\check{y}})$, the fluid velocity as $(u,v,w)=V({\check{u}},{\check{v}},\delta {\check{w}})$, the pressure as $p={\check{p}}{\mu V L}/{z_w^2}$, and time as $t={\check{t}}{L}/{V}$; here, *L* is a characteristic lateral extent, *V* is a scale for the velocity to be defined later, $\mu$ is the fluid viscosity, and $\delta \equiv z_w/L \ll 1$ is a small parameter, which will be used as the basis for the asymptotic reduction to be carried out below. The magnetic film in each layer is scaled as $\mathbf{H}_i=H_\infty {\check{\mathbf{H}}}_i$, the magnetic potential as $\phi _i={H_\infty }{z_w}{\check{\phi }}_i$, and the magnetization as $\mathbf{M}_i= H_\infty {\check{\mathbf{M}}}_i$, where $i=1$ and 2 for the film and gas, respectively, and $H_\infty$ is the incident magnetic field. Henceforth, we drop the check decoration for the dimensionless quantities.

In the film (contained in a domain defined by $-1 \le z\le S$) and surrounding gas ($z\le S$), the magnetic field can be defined in terms of the potential as $\mathbf{H}_i=-(\delta \phi _{ix},\delta \phi _{iy},\phi _{iz})$, where the magnetic potential, $\phi _i$, is determined from

1 $$\begin{aligned}&\left( (1+\beta F){\phi _1}_{z}\right) _z+ \delta ^2\left( (1+\beta F){\phi _1}_{x}\right) _x+\delta ^2\left( (1+\beta F){\phi _1}_{y}\right) _y=0,\,\,\, -1 \le z\le S, \,\,\,\,\,\,\,\,\,\, \end{aligned}$$

2 $$\begin{aligned}&\phi _{2zz}+\delta ^2\left( \phi _{2xx}+\phi _{2yy}\right) =0, \,\,\, z \ge S, \end{aligned}$$

in the film and surrounding gas, respectively. The parameter $\beta F$ is related to the dimensionless magnetization defined from the relationship

3 $$\begin{aligned} \mathbf{M}_1=\beta \left( \coth ({\xi '} H_1)-\frac{1}{{\xi '} H_1} \right) \frac{\mathbf{H}_1}{H_1}=\beta F(H_1)\mathbf{H}_1, \end{aligned}$$

where $\beta =M_s/H_\infty , {\xi '}=\xi H_\infty =3\chi _0H_{\infty }/M_s, \chi _0$ is the initial susceptibility, $M_s$ is the saturation magnetization, $H_i=(\phi _{iz}^2+\delta ^2\phi _{ix}^2+\delta ^2\phi _{iy}^2)^{1/2}, M_i=\beta F (\phi _{iz}^2+\delta ^2\phi _{ix}^2+\delta ^2\phi _{iy}^2)^{1/2}$.

For the free surface, $z=S(x,y)$, we define the normal and tangential vectors

4 $$\begin{aligned} \mathbf{n}=\frac{(-\delta S_x,-\delta S_y,1)}{\sqrt{1+\delta ^2 S_x^2 +\delta ^2 S_y^2}},\quad \mathbf{t}_x=\frac{(1,0,\delta S_x)}{\sqrt{1+\delta ^2 S_x^2}},\quad \mathbf{t}_y=\frac{(0,1,\delta S_y)}{\sqrt{1+\delta ^2 S_y^2}}, \end{aligned}$$

as well as the normal and tangential jump conditions from Eqs. (1) and (2) in dimensionless form:

5 $$\begin{aligned}&(1+\beta F)\left( \phi _{1z}-\delta ^2S_{x} \phi _{1x}-\delta ^2S_{y} \phi _{1y}\right) = \left( \phi _{2z}-\delta ^2S_{y} \phi _{2y}-\delta ^2S_{x} \phi _{2x}\right) , \end{aligned}$$

6 $$\begin{aligned}&S_{x}\phi _{1z}+\phi _{1x}=S_{x}\phi _{2z}+\phi _{2x},\end{aligned}$$

7 $$\begin{aligned}&S_{y}\phi _{1z}+\phi _{1y}=S_{y}\phi _{2z}+\phi _{2y}. \end{aligned}$$

At the wall, $z=-1$, we fix the magnetic field as was done by [27], which is different from the model of [6]. The appropriate condition in this case for a flat surface is

8 $$\begin{aligned} (1+\beta F)\phi _{1z}=H_w=\delta {\bar{H}}_w, \end{aligned}$$

where we have set the substrate magnetic field to a small value so that it appears in the leading-order terms to be discussed in the next section. Far from the film, the magnetic field is $H_2 \rightarrow {H}_\infty$ as $z\rightarrow + \infty$.

The dimensionless momentum and continuity equations are

9 $$\begin{aligned}&Re\,\delta \left( {u}_{t}+w{u}_{z}+u{u}_{x}+v{u}_{y}\right) =-{p}_{x} + \delta Q_H {\varOmega }_{x}+\nabla ^2 u +G, \end{aligned}$$

10 $$\begin{aligned}&Re\,\delta \left( {v}_{t}+w{v}_{z}+u{v}_{x}+v{v}_{y}\right) =-{p}_{y} + \delta Q_H {\varOmega }_{y} +\nabla ^2 v, \end{aligned}$$

11 $$\begin{aligned}&Re\,\delta ^3\left( {w}_{t}+w{w}_{z}+u{w}_{x}+v{w}_{y}\right) =-{p}_{z} +\delta Q_H {\varOmega }_{z}+\delta ^2 \nabla ^2 w -\delta G \cot (\theta ), \end{aligned}$$

12 $$\begin{aligned}&{w}_{z}+{u}_{x}+{v}_{y}=0, \end{aligned}$$

where $\nabla ^2=\partial ^2_z + \delta ^2(\partial ^2_x+\partial ^2_y)$, and the dimensionless Maxwell pressure reads

13 $$\begin{aligned} \varOmega =-\frac{\beta }{{\xi '}}\ln \left( \frac{{H}{\xi '}}{\sinh ({H}{\xi '})} \right) . \end{aligned}$$

Here, the dimensionless groups appearing in the momentum equation are defined as

14 $$\begin{aligned} Re=\frac{\rho V z_w}{\mu }, \qquad Q_H=\frac{\mu _0 H_\infty ^2 z_w}{\mu V}, \qquad G=\frac{\rho g z_w^2 \sin {\theta }}{\mu V}, \end{aligned}$$

and represent the Reynolds number wherein $\rho$ is the fluid density, a magnetic parameter in which $\mu _0$ (not to be confused with the viscosity) is the magnetic permeability, and a gravitational parameter, respectively.

At the interface, the normal and tangential stress balances are

15 $$\begin{aligned} \left\| \mathbf{n}\cdot {\mathbf{T}}^f\cdot \mathbf{t}\right\|= & {} 0 , \end{aligned}$$

16 $$\begin{aligned} \left\| \mathbf{n}\cdot {\mathbf{T}}^f\cdot \mathbf{n}\right\|= & {} -Q_H\delta \frac{1}{2}(\mathbf{M}\cdot \mathbf{n})^2+\delta ^3Ca^{-1}\,{{\mathcal {K}}}, \end{aligned}$$

where $Ca=\mu V/\gamma$ is the capillary number, $\gamma$ is the surface tension, and ${{\mathcal {K}}}$ is the curvature of the interface:

17 $$\begin{aligned} {{\mathcal {K}}}=-\frac{(1+\delta ^2 S_y^2)S_{xx}-2\delta ^2S_xS_xS_{xy}+(1+\delta ^2S_x^2)S_{yy}}{(1+\delta ^2S_x^2+\delta ^2S_y^2)^{3/2}}, \end{aligned}$$

and the fluid stress is

18 $$\begin{aligned} \mathbf{T}^f=-p\mathbf{I}+\frac{1}{2}\delta \frac{\mu _i}{\mu _w}\left( (\nabla \mathbf{u})+(\nabla \mathbf{u})^T\right) . \end{aligned}$$

Finally, the dimensionless kinematic condition is

19 $$\begin{aligned} S_{t}+uS_{x}+vS_{y}=w, \end{aligned}$$

and, at the substrate surface, $z=-1, u=v=w=0$ corresponding to the no-slip and no-penetration conditions.

### Long-wave approximation

We seek a solution to the magnetic potential for a large effective magnetic permeability of the ferrofluid. In this way, the film, as viewed from the outer field, resembles a magnetized sheet. With this assumption, we take the order of magnitude of the dimensionless groups as $\beta =O(1)$ and ${\xi '}=O(1)-O(10)$ as discussed in [6]. The surrounding gas region is not slender so we re-scale as $z=\tilde{z}\delta ^{-1}$ [19]. We now set the velocity scale to $V=\delta ^3\gamma /\mu$ so that the capillary number in Eq. (16) is $Ca=\mu V/\gamma =\delta ^3$, and the capillary pressure term in this equation is of order unity. We also set ${\bar{Q}}_H=\delta ^2 Q_H$ so the Maxwell pressure is of the same order as the capillary pressure. In addition, we take ${\xi '}={\bar{\xi }}/\delta , F={\bar{F}}\delta ^{-1}$, and $H_\infty =1+\delta H_1(x)$. Therefore, the magnetization term is expanded as $(1+\beta F)=\delta ^{-1}(\delta +\beta {\bar{F}})$ and Eqs. (1) and (2) for the magnetic potential in the ferrofluid film and gas, respectively, become

20 $$\begin{aligned}&\left( (\delta +\beta {\bar{F}} ) \phi _{1z} \right) _z+\delta ^2\left( (\delta +\beta {\bar{F}} ) \phi _{1x} \right) _x +\delta ^2\left( (\delta +\beta {\bar{F}} ) \phi _{1y} \right) _y =0, \quad -1 \le z\le S, \end{aligned}$$

21 $$\begin{aligned}&\phi _{2 \tilde{z}\tilde{z}}+\phi _{2xx}+\phi _{2yy}=0, \quad \tilde{z} \ge \delta S. \end{aligned}$$

The far-field boundary condition is

22 $$\begin{aligned} \phi _{2\tilde{z}}= & {} {H}_{\infty }/\delta , \quad \tilde{z}\rightarrow + \infty , \end{aligned}$$

and the interfacial conditions corresponding to Eqs. (5), (6) are expressed as

23 $$\begin{aligned}&(\delta +\beta {\bar{F}})\left( \phi _{1z}-\delta ^2 S_x\phi _{1x}-\delta ^2 S_y\phi _{1y}\right) |_S = \delta ^2 \left( \phi _{2\tilde{z}}-\delta S_x\phi _{2x}-\delta S_y\phi _{2y} \right) |_{\delta S}, \end{aligned}$$

24 $$\begin{aligned}&(S_{x}\phi _{1z}+\phi _{1x})|_S = (\delta S_{x}\phi _{2\tilde{z}}+\phi _{2x})|_{\delta S},\end{aligned}$$

25 $$\begin{aligned}&(S_{y}\phi _{1z}+\phi _{1y})|_S = (\delta S_{y}\phi _{2\tilde{z}}+\phi _{2y})|_{\delta S}. \end{aligned}$$

The substrate conditions corresponding to Eqs. (7), (8) are now re-written as

26 $$\begin{aligned} (\delta+ & {} \beta {\bar{F}})\phi _{1z}|_{-1}=\delta ^2 {\bar{H}}_w, \,\,\,\,\,\,\, \end{aligned}$$

where the solid wall is at $z=-1$.

We now expand the potential in a perturbation series of the form $\phi _{i}=\phi _{i0}+\delta ^2 \phi _{i1}+\cdots$ (for $i=1$ and 2), which is asymptotically valid in the limit of $\delta \rightarrow 0$, and collect terms of equal powers of $\delta$. The idea is that we are determining the linear solution and its perturbation in powers of a small parameter $\delta ^2$ (for more details, see Hinch [28]). From Eq. (20), the potential to leading-order (i.e. the equation with order $\delta ^0$) is $\beta {\bar{F}}\phi _{10z}=c(x,y)$ in the film, where $c(x,y)=0$ from the boundary conditions. Integrating again, the potential is only a function of *x* and *y*, $\phi _{10}=\phi _{10}(x,y)$, and is determined from the next order of the expansion. Also to leading-order $\phi _{10}|_S=\phi _{20}|_{\delta S}$ from Eq. (24), $|\mathbf{H}_1|=\delta (\phi _{10x}^2+\phi _{10y}^2)^{1/2}$ and

27 $$\begin{aligned} {\bar{F}}=\left( \frac{\coth {({\bar{\xi }}(\phi _{10x}^2+\phi _{10y}^2)^{1/2})}}{(\phi _{10x}^2+\phi _{10y}^2)^{1/2}}-\frac{1}{{\bar{\xi }}(\phi _{10x}^2+\phi _{10y}^2) } \right) , \end{aligned}$$

which is only a function of *x* and *y*.

For the order $\delta ^2$ equation, the potential in the film to the next order is determined from the following set of equations:

28 $$\begin{aligned}&\phi _{11zz}=-\frac{1}{{\bar{F}}}{\left( {\bar{F}}\phi _{10x}\right) }_{x}-\frac{1}{{\bar{F}}}{\left( {\bar{F}}\phi _{10y}\right) }_{y},\end{aligned}$$

29 $$\begin{aligned}&\beta {\bar{F}}\left( \phi _{11z}- S_x\phi _{10x}- S_y\phi _{10y}\right) |_S= \left( \phi _{20\tilde{z}} \right) |_{\delta S}, \end{aligned}$$

30 $$\begin{aligned}&\beta {\bar{F}}\phi _{11z}|_{-1}= {\bar{H}}_w. \end{aligned}$$

Integrating Eq. (28) once with respect to *z*, inserting into the above boundary conditions, and subtracting we get

31 $$\begin{aligned} \phi _{20\tilde{z}} |_{\delta S}-{\bar{H}}_w= & {} -\beta {\bar{F}}\left( S_x\phi _{10x}+S_y\phi _{10y}\right) -\,\beta {\bar{F}}\left( \frac{1}{{\bar{F}}}{\left( {\bar{F}}\phi _{10x}\right) }_{x}(1+S)+\frac{1}{{\bar{F}}}{\left( {\bar{F}}\phi _{10y}\right) }_{y}(1+S)\right) , \end{aligned}$$

which is a condition for the horizontal gradient of the potential.

The potential in the outer gas is more easily determined through subtracting the outer magnetic field by defining $\phi _{20\tilde{z}}=\varPhi _{20\tilde{z}}+H_\infty /\delta$. The equations for the new variable are

32 $$\begin{aligned} \varPhi _{20\tilde{z}\tilde{z}}+\varPhi _{20xx}+\varPhi _{20yy}=0, \quad \tilde{z} \ge \delta S, \end{aligned}$$

with the following far-field and interfacial conditions, respectively, given by

33 $$\begin{aligned}&\varPhi _{20\tilde{z}}=0 ,\quad \tilde{z}\rightarrow + \infty , \end{aligned}$$

34 $$\begin{aligned}&\varPhi _{20}|_{\delta S}=\phi _{10}|_{S} -H_\infty S. \end{aligned}$$

We solve Laplace’s equation in the gas phase by using the Fourier transform defined as

35 $$\begin{aligned} {\hat{\phi }}({\hat{k}}_x,{\hat{k}}_y)= & {} \int _{-\infty }^{\infty }\int _{-\infty }^{\infty }\varPhi (x,y,z) \, \mathrm{e}^{\mathrm{i} ({\hat{k}}_x x+{\hat{k}}_y y)} \, \mathrm {d}x\,\mathrm {d}y, \end{aligned}$$

36 $$\begin{aligned} \varPhi (x,y,z)= & {} \frac{1}{2 \pi }\int _{-\infty }^{\infty }\int _{-\infty }^{\infty }{\hat{\phi }}\big ({\hat{k}}_x,{\hat{k}}_y \big )\, \mathrm{e}^{-\mathrm{i} ({\hat{k}}_x x+{\hat{k}}_y y)} \, \mathrm {d}{\hat{k}}_x \,\mathrm {d}{\hat{k}}_y, \end{aligned}$$

where ${\hat{k}}_x$ and ${\hat{k}}_y$ are the wavenumbers in the *x* and *y*-directions, respectively. Here, the integrations are taken to infinity in *x* and *y*; however, it is possible to integrate the function $\varPhi$ over a finite domain provided it is periodic at the boundaries. In this analysis, we assume that the surface height, velocity, pressure and magnetic potential have periodic boundary conditions in the *x*–*y* plane, and perform the integrations over a finite domain. Laplace’s equation in Fourier space becomes

37 $$\begin{aligned} {\hat{\phi }}_{2\tilde{z}\tilde{z}} -{\hat{k}}^2{\hat{\phi }}_2=0, \end{aligned}$$

where $${\hat{k}}^2={\hat{k}}_x^2+{\hat{k}}_y^2$$.

Using the boundary conditions far from the film, $\varPhi _{2\tilde{z}}=0$ as $\tilde{z}\rightarrow + \infty$, and the condition given by Eq. (34), the potential in spectral space is

38 $$\begin{aligned} {\hat{\phi }}_2=\left( {\hat{\phi }}_{10}-\widehat{S H_\infty } \right) \mathrm{e}^{-{\hat{k}}\tilde{z}}, \end{aligned}$$

where $\widehat{S H_\infty }$ is the Fourier transform of the product of *S* and $H_\infty$ and the derivative of Eq. (38) at $\delta S=0$ is

39 $$\begin{aligned} {\hat{\phi }}_{2\tilde{z}}=-{\hat{k}} \left( {\hat{\phi }}_{10}-\widehat{S H_\infty } \right) . \end{aligned}$$

In the dimensionless momentum equations, we take $Re=O(1)$ and expand in powers of $\delta$ (e.g. $u=u_0+\delta u_1+\cdots$). The continuity and momentum equations to leading order are

40 $$\begin{aligned} u_{0x}+ & {} v_{0y}+w_{0z}=0, \end{aligned}$$

41 $$\begin{aligned} p_{0z}= & {} {\bar{Q}}_H \varOmega _{0z} -{\hat{G}}, \end{aligned}$$

42 $$\begin{aligned} p_{0x}= & {} {\bar{Q}}_H \varOmega _{0x}+ u_{0zz} +G, \end{aligned}$$

43 $$\begin{aligned} p_{0y}= & {} {\bar{Q}}_H \varOmega _{0y}+ v_{0zz}, \end{aligned}$$

where $${\hat{G}}=\delta G \cot (\theta )$$ and

44 $$\begin{aligned} \varOmega _0=- \frac{\beta }{ {\bar{\xi }}} \ln \left( \frac{(\phi _{10x}^2+\phi _{10y}^2)^{1/2}{\bar{\xi }}}{\sinh ((\phi _{10x}^2+\phi _{10y}^2)^{1/2}{\bar{\xi }})} \right) . \end{aligned}$$

At the interface $z=S(x,y)$, the normal and tangential jump conditions to leading-order are

45 $$\begin{aligned}&p_0=-\frac{{\bar{Q}}_H}{2}\left( \mathbf{H}_{20}\cdot \mathbf{n}-\mathbf{H}_{10}\cdot \mathbf{n}\right) ^2 - (S_{xx}+S_{yy}),\end{aligned}$$

46 $$\begin{aligned}&u_{0z}=0,\end{aligned}$$

47 $$\begin{aligned}&v_{0z}=0. \end{aligned}$$

where $\left( \mathbf{H}_{20}\cdot \mathbf{n}-\mathbf{H}_{10}\cdot \mathbf{n}\right) ^2=\delta ^{-1}+2(H_1(x)+\varPhi _{20\tilde{z}})$ and $\delta$ have been retained here for completeness; however, it will be eliminated later when we take the derivative of this quantity. Integrating the leading-order momentum equations, and using the interfacial (air–film interface located at $z=S(x,y,t)$) and no-slip boundary conditions at $z=-1$, yields the following expressions for the film pressure, and streamwise and spanwise velocity components, respectively:

48 $$\begin{aligned} p_0= & {} {\bar{Q}}_H \varOmega _0-{\hat{G}} (z+1)+K(x,y),\end{aligned}$$

49 $$\begin{aligned} u_0= & {} (K_{x}-G) \left( \frac{1}{2}(z+1)^2-(S+1)(z+1) \right) ,\end{aligned}$$

50 $$\begin{aligned} v_0= & {} K_{y} \left( \frac{1}{2}(z+1)^2-(S+1)(z+1) \right) . \end{aligned}$$

From the kinematic condition, the interface evolves according to

51 $$\begin{aligned} S_t-\frac{1}{3}\left[ (S+1)^3 (K_x-G) \right] _x-\frac{1}{3}\left[ (S+1)^3 K_y \right] _y=0, \end{aligned}$$

where the two-dimensional function *K*(*x*, *y*) is

52 $$\begin{aligned} K(x,y)= & {} {\bar{Q}}_H\left( \frac{\beta }{ {\bar{\xi }}} \ln \left( \frac{(\phi _{10x}^2+\phi _{10y}^2)^{1/2}{\bar{\xi }}}{\sinh ((\phi _{10x}^2+\phi _{10y}^2)^{1/2}{\bar{\xi }})} \right) -\frac{1}{2}\delta ^{-1}\right) -{\bar{Q}}_H\left( (H_1(x)+\varPhi _{20\tilde{z}}) \right) - S_{xx} -S_{yy}+{\hat{G}} S. \end{aligned}$$

Here, we have defined $H_\infty =1+\delta H_1(x)$. Note that the presence of the $O(\delta ^{-1})$ in the expression for *K*(*x*, *y*) is inconsequential since $K_x$ and $K_y$ (rather than *K*(*x*, *y*)) are required to form *u* and *v*, respectively. In Eq. (52), the vertical derivative of the magnetic potential is

53 $$\begin{aligned} \varPhi _{20\tilde{z}}(x,y,z)=\frac{1}{2 \pi }\int _{-\infty }^{\infty }\int _{-\infty }^{\infty } {\hat{k}} \left( \widehat{S H_\infty }- {\hat{\phi }}_{10} \right) \, \mathrm{e}^{-\mathrm{i} ({\hat{k}}_x x+{\hat{k}}_y y)} \, \mathrm {d}{\hat{k}}_x \mathrm {d}{\hat{k}}_y, \end{aligned}$$

where the potential ${\hat{\phi }}_{10}$ comes from Eq. (31) transformed to Fourier space.

## Results

The governing equations describing the flow of a thin ferrofluid in a magnetic field are given by Eqs. (31), (39), (51), and (52). The dimensionless groups governing the process are: the saturation magnetization relative to the outer magnetic field, $\beta$, the magnetic parameter, ${\bar{Q}}_H$, the scaled initial susceptibility, ${\bar{\xi }}$, and the gravitational numbers *G*, and ${\hat{G}}=\delta G \cot (\theta )$. For typical laboratory conditions, using magnetite as the suspended particles, we can assume the following parameter values: $M_s\approx 1.5 \times 10^4~\mathrm{A}/\mathrm{m}, \xi \approx 2\times 10^{-4}-5\times 10^{-4}~\mathrm{m}/\mathrm{A}$, and $H_{\infty }\sim M_s$ [14]. The order of magnitude for the dimensionless groups are then: $\beta =O(1), {{{\bar{\xi }}}}=O(1)-O(10)$, and ${\bar{Q}}_H=O(1)$. The effect of the magnetic and fluid dynamics parameters were studied by [6]. Here, we focus on the magnetic field strength ${\bar{Q}}_H$, and fix the other parameters to $\beta =1, {{{\bar{\xi }}}}=5, G=0.50$, and ${\hat{G}}=0.25$.

### Linear stability analysis

It is instructive to analyse the linear stability for a flat interface in order to gain insight into the dynamics of a ferrofluid film near instability onset. We do this by linearizing the governing equations about the uniform base state $S=0$ and $\phi =\phi _b$ (a constant) using normal modes of the form:

54 $$\begin{aligned} S=S'={\bar{S}} \, \mathrm{e}^{\omega t +\mathrm{i}k_xx+\,\mathrm{i}k_yy}, \quad \phi -\phi _b=\phi '={\bar{\phi }}\, \mathrm{e}^{\omega t+\mathrm{i}k_xx+\,\mathrm{i}k_yy}. \end{aligned}$$

Here, $k_x$ and $k_y$ are the wavenumbers in the *x* and *y*-directions, respectively; ${\bar{S}}$ and ${\bar{\phi }}$ are constants to be determined below; and $S'$ and $\phi '$ are the perturbations about the base state. Introducing the expansions into Eqs. (31) and (39) and retaining terms on the order of the perturbation, the magnetic potential is expressed as

55 $$\begin{aligned} {\bar{\phi }}=\frac{{\bar{S}}-H_w/k}{1+k\beta {\bar{\xi }}/3 }, \end{aligned}$$

with the magnetic potential being linearly related to the interface height and $k^2=k_x^2+k_y^2$. We take $H_w=0$ for simplicity, and from Eqs. (51) and (52), the real part for the dispersion relation is obtained as

56 $$\begin{aligned} 3\omega _r ={\bar{Q}}_H k^3 \left( 1-\frac{1}{1+k\beta {\bar{\xi }}/3} \right) -k^4 - {\bar{G}} k^2 . \end{aligned}$$

**Fig. 1:**
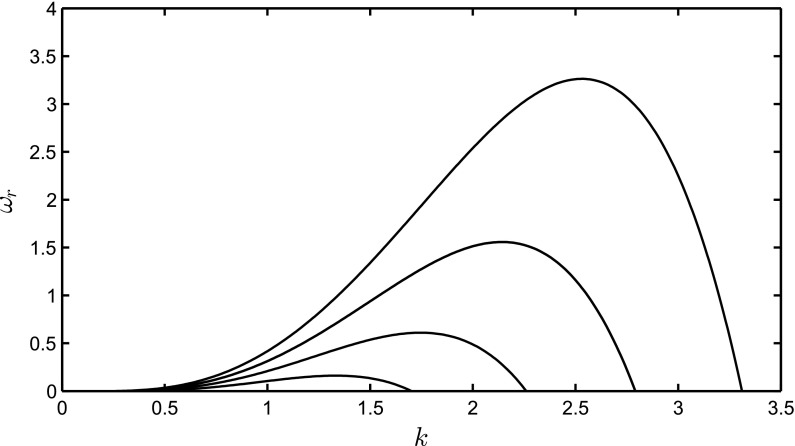
Dispersion relations for ${\bar{Q}}_H=2.5, 3, 3.5, 4$, corresponding to the lowest to highest curves, respectively. The rest of the parameters are ${\bar{\xi }}=5, \beta =1, G=0.0$, and ${\hat{G}}=0.25$

The first term is positive so the magnetic field is destabilizing, whereas capillary forces always stabilize the film for sufficiently large *k* as found by [6, 11, 29]. The dispersion relation, for $k_y=0$ and $k_x=k$, is displayed in Fig. 1, showing the wavenumber band for instability. The result is similar to the electrohydrodynamic case [17], where the electric field, for a perfectly conducting viscous film, was also found to be destabilizing.

**Fig. 2:**
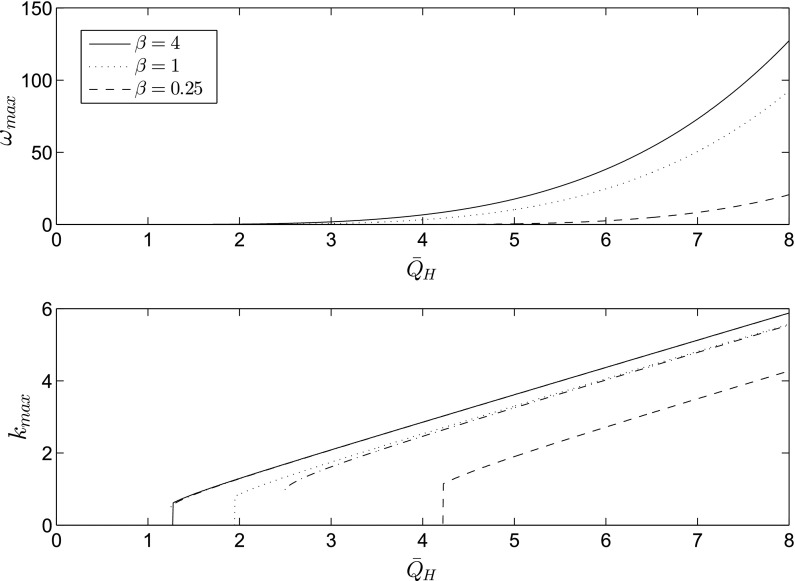
Maximum growth rate, $\omega _{\mathrm{max}}$, and the most dangerous mode, $k_{\mathrm{max}}$, as a function of ${\bar{Q}}_H$; the *dash-dot line* corresponds to Eq. (57). The remaining parameters are ${\bar{\xi }}=5, G=0.0$, and ${\hat{G}}=0.25$

In the large $\beta {\bar{\xi }} k$ limit, we simplify the dispersion relation as $\omega ={\bar{Q}}_Hk^3/3-{\bar{Q}}_Hk^2/\beta {\bar{\xi }}-k^4/3-{\bar{G}}k^2/3$. Taking the derivative of this equation with respect to *k* and setting it to zero, we obtain the location of the maximum growth rate as

57 $$\begin{aligned} k_\mathrm{max}=\frac{3}{8}{\bar{Q}}_H+\frac{1}{2}\sqrt{\frac{9}{16}{\bar{Q}}^2_H-6\frac{{\bar{Q}}_H}{\beta {\bar{\xi }}} +2{\bar{G}} }. \end{aligned}$$

Since we only want the real part of the expression, the jump occurs at

58 $$\begin{aligned} {\bar{Q}}_H=\frac{16}{3}\frac{1}{\beta {\bar{\xi }}}+\frac{1}{2}\sqrt{\left( \frac{32}{3}\frac{1}{\beta {\bar{\xi }}}\right) ^2+\frac{128}{9}{\bar{G}}}. \end{aligned}$$

In Fig. 2, the maximum growth rate and the most dangerous mode are shown as a function of ${\bar{Q}}_H$ and $\beta$. The most dangerous mode exhibits a jump followed by a linear increase with ${\bar{Q}}_H$. The large $\beta {\bar{\xi }} k$ solution from Eq. (57) is in close agreement with the full solution even for moderate values of $\beta$. The prediction of the jump in $k_\mathrm{max}$ from Eq. (58) is also very good for sufficiently large $\beta {\bar{\xi }} k$.

### Numerical results

We solve the governing equations using pseudo-spectral methods with a sufficiently large number of modes in the *x*–*y* direction. Typically, we use $256 \times 64$ modes in the simulations without any discernible difference in the results with half or double the number of modes. Two-dimensional Fast Fourier Transforms are used for the basis functions and the boundary conditions are periodic. The thin film equations are discretized in time using an explicit RK-3 method, and the magnetic field equations are solved using an iterative method. Domain decomposition is used to parallelize the code using MPI-2. With this technique, the spatial domain is broken up into strips along the *y*-direction with each processor computing $n_y/n_p\times n_x$ points, where $n_y$ is the number of modes in the *y*-direction, $n_p$ the number of processors and $n_x$ the number of modes in the *x*-direction. The main MPI communications occur in the FFT routines in order to perform the two-dimensional integrations. We checked that mass conservation is maintained in the computations and that the solution remains unchanged with the increasing resolution. A precursor film is used to avoid the contact line stress singularity with a thickness that is sufficiently small. For an initial profile, we use a Gaussian distribution: $S(x,y,0)=S_f+\exp (-(x-x_0)^2/3)$. Here, $S_f=-0.98$ (the precursor film thickness is then 0.02) is the location of the precursor film, and we take $x_0=5$ unless otherwise specified. Given the initial surface profile, we obtain the initial magnetic potential by solving Eq. (31). In addition, we use periodic boundary conditions at the ends of the *x*–*y* domain for *S*(*x*, *y*, *t*) and $\phi _{10}(x,y,t)$.

**Fig. 3:**
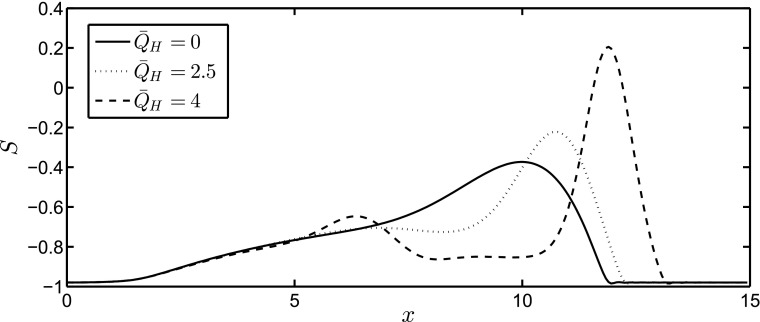
The parametric dependence of the spatial development of the interface *S* on ${\bar{Q}}_H$ for the two-dimensional case in the absence of spanwise perturbations at a dimensionless time of $t=80$. Here, ${\bar{\xi }}=5, \beta =1, G=0.5$, and ${\hat{G}}=0.25$

**Fig. 4:**
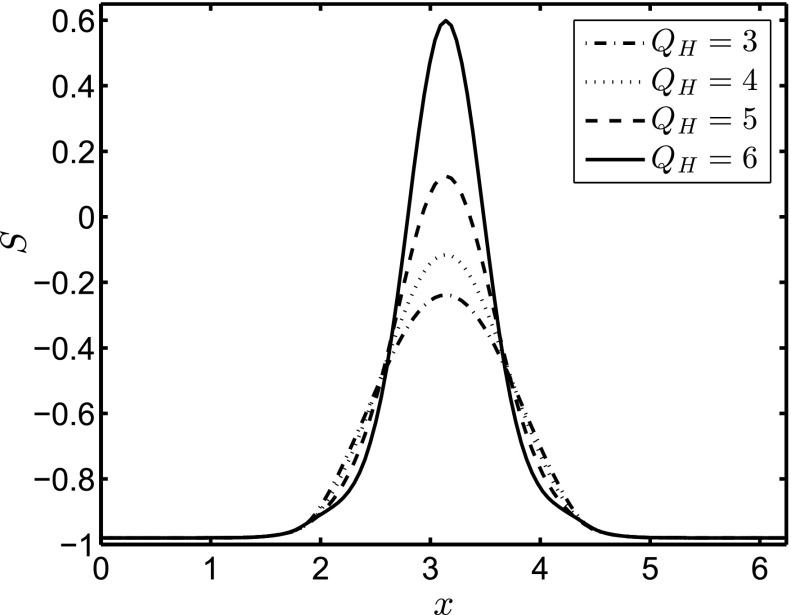
Parameteric dependence of the steady-state value of *S* on ${\bar{Q}}_H$ for a three-dimensional axisymmetric drop spreading on a flat substrate in the presence of a magnetic field. Here, ${\bar{\xi }}=5, \beta =1$, and $G=0.0={\hat{G}}=0.0$. We also use the same initial Gaussian profile for the surface height with $x_0=\pi$

#### Two-dimensional unperturbed case

First, we investigate the case of a two-dimensional (2D) flow in the absence of imposed spanwise perturbations, which will be used in the subsequent stability analysis to be discussed below. In Fig. 3, we show snapshots of the spatial development of the interface *S* at $t=80$ for three different values of ${\bar{Q}}_H$, including the non-magnetic case, with ${\bar{\xi }}=5, \beta =1, G=0.5$, and ${\hat{G}}=0.25$ held constant. For all values of ${\bar{Q}}_H$ considered (including ${\bar{Q}}_H=0$, as expected), the drop forms a capillary ridge near the advancing ‘contact line’ located at the downstream leading edge, and a long tail which extends back to the initial drop location. The magnetic field creates a jump in $\phi _{10x}$, causing an increased pressure at the peak. As a result, the drop accelerates and the capillary ridge steepens and increases in height. In addition, for the largest values of ${\bar{Q}}_H$ examined, a ‘secondary’ ridge appears in the tail region with a trough forming between this and the ‘primary’ ridge at the leading edge. To isolate the effect of ${\bar{Q}}_H$ (from those associated with the gravitational acceleration) on the primary ridge structure, we examine briefly the case of a drop on a flat substrate in the presence of a magnetic field. The drop structure is initialized by the same Gaussian profile as that used to generate the results in Fig. 3, and the simulation is carried out until a steady-state solution is reached. As shown in Fig. 4, for increasing values of ${\bar{Q}}_H$, the peak height of the drop, $S_{\mathrm{max}}$, increases, and the drop narrows due to mass conservation, forming spike-like structures.

**Fig. 5:**
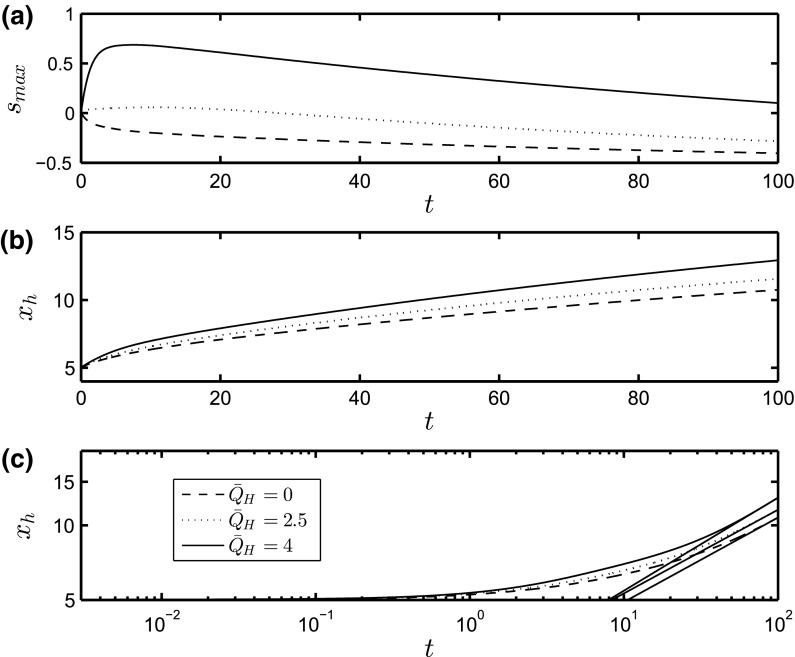
Temporal evolution of the maximal interface height, $S_{\mathrm{max}}$, (**a**) and its spatial location, $x_h$, (**b**) for ${\bar{Q}}_H=0,2.5,4$. A log-log plot of $x_h$ vs *t* is shown in (**c**). The *straight solid lines in panel* (**c**) correspond to a power-law of the form $x_h=at^n$, where $n \sim (0.34, 0.34, 0.38),$ and $a \sim (2.24, 2.44, 2.29)$ for ${\bar{Q}}_H=0,2.5, 4,$ respectively. The rest of the parameters remain unchanged from Fig. 3

The time evolution of the maximum height of the drop, $S_{\mathrm{max}}$, and that of the spatial location corresponding to $S_{\mathrm{max}}, x_h$, are plotted in Fig. 5. For the non-magnetic case, ${\bar{Q}}_H=0, S_{\mathrm{max}}$ decreases with time starting from $t=0$ as it spreads down the inclined plane. When the magnetic pressure is applied, i.e. for ${\bar{Q}}_H > 0, S_{\mathrm{max}}$ increases initially as the surface is pulled in the direction of the incident magnetic field but later decreases as the constant volume drop flows down the incline. It is also evident from Fig. 5 that the spreading is accelerated via the application of the magnetic field through inspection of the temporal evolution of $x_h$ which increase with ${\bar{Q}}_H$ for all *t* values considered.

A semilogarithmic plot of $x_h$ vs. *t* shown in Fig. 3c reveals that the spreading of the drop exhibits a power-law behaviour with a well-defined exponent at large times. In all of the cases shown, the exponent is similar for different values of ${\bar{Q}}_H$ although the slope is a little steeper for larger vales of the magnetic pressure. It appears from the power-law exponent that the motion of the ridge down the incline is dominated by gravity despite the presence of the magnetic field for all the ${\bar{Q}}_H$ investigated. Neglecting magnetic and capillary effects in Eq. (51), and taking into consideration spreading in the $x-$direction only, we have $S_t + \left[ G\left( S+1)^3\right) \right] /3 \sim 0$. The continuity constraint then leads to $x_h \sim t^{1/3}$, which is in agreement with the numerically determined exponents (see Fig. 5).

#### Single spanwise mode

We now examine the stability of the 2-D states discussed in the foregoing. We perform the stability analysis by perturbing a 2-D simulation at time $t_p$, which occurs at a time where the front is sufficiently developed. The front position is perturbed by applying a spanwise disturbance, which is added to the right-hand-side of Eq. (51) expressed by

59 $$\begin{aligned} f_p=A_0\cos (k_p y)G_a(t)S_x, \end{aligned}$$

where $A_0$ is the amplitude, $k_p$ is the wavenumber, and $G_a(t)$ is a Gaussian function of time that is centred at $t=t_p$. In the absence of perturbations, the front is stable for very long times, as has been verified numerically. The simulations are performed in a moving reference at the speed $v_f$, which was shown by [22] not to affect the results. We also note that in this case, the front is stable to spanwise perturbations in the absence of a Maxwell pressure.

**Fig. 6:**
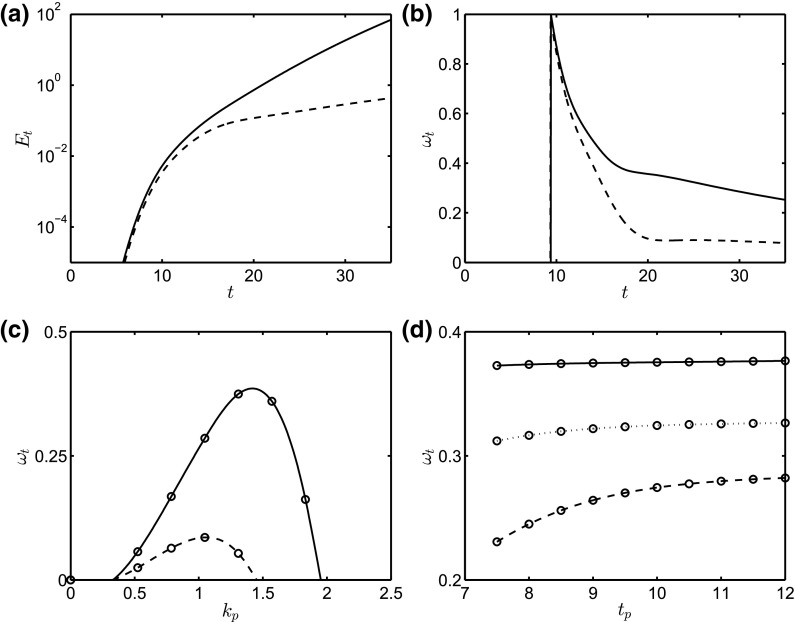
Stability of the two-dimensional solutions to spanwise disturbances. Temporal evolution of the disturbance ‘energy’, $E_t$, and growth rate, $\omega _t$, are shown in (**a**, **b**), respectively, for ${\bar{Q}}_H=2.5$ (*dashed line*) and ${\bar{Q}}_H=3.0$ (*solid line*). Numerically constructed ‘dispersion relations’ for ${\bar{Q}}_H=2.5$ (*dashed line*), and ${\bar{Q}}_H=3$ (*solid line*) are shown in (**c**) for $t=30$. The parametric dependence of $\omega _t$ on $t_p$ for ${\bar{Q}}_H=3.0$, and $t=25$ (*solid line*), $t=30$ (*dotted line*), and $t=35$ (*dashed line*) is shown in (**d**). In (**c**) and (**d**), the *circles* correspond to the numerical solution, and the *lines* represent the interpolated values. Here, $A_0=1\times 10^{-3}$ (see Eq. (59)); the rest of the parameters remain unchanged from Fig. 3

In order to avoid the complications associated with the temporally evolving base state in the linearized form of the governing equations, we determine the rate of growth of the spanwise stability numerically. To do this, we define a scaled spanwise ‘energy’, $E_t$, and a corresponding growth rate, $\omega _t$, as

60 $$\begin{aligned} E_t=\frac{\int S_y^2(x,y,t)\mathrm {d}x\, \mathrm {d}y}{\int S_y^2(x,y,t_p)\mathrm {d}x\, \mathrm {d}y},\qquad \omega _t=\frac{1}{E_t}\frac{\partial E_t}{\partial t}. \end{aligned}$$

This expression is similar to that used by [30], except that we are using the derivative of the interface in the spanwise direction $S_y$ to represent the growth in energy of the perturbation. It is important to note that our approach does not correspond to a linear stability analysis: we have not linearized Eq. (51), and indeed the amplitude of the imposed perturbation given by Eq. (59), although initially small, is permitted to grow such that the disturbance can enter into the nonlinear regime. Thus, $\omega _t$ does not correspond to the growth rates that can be obtained from a linear stability analysis (except perhaps over a limited time duration in which the perturbation has grown exponentially in amplitude yet its amplitude has remained sufficiently small so that we are still in the linear regime).

The values of $E_t$ and $\omega _t$ are tracked during the course of the simulation from $t_p$ onwards for a given value of $k_p$, as shown in Fig. 6a, b. It is seen clearly that $E_t$ and $\omega _t$ undergo an increase with time for $t>t_p$ indicating disturbance growth, with both growth measures increasing with ${\bar{Q}}_H$, which highlights the destabilizing effect of the imposed magnetic field. From Fig. 6b, it is evident that following the initial rapid increase, the disturbance growth rate decreases at later times; in the case of ${\bar{Q}}_H=2.5, \omega _t$ appears to asymptote to a constant value.

In Fig. 6c, we plot numerically constructed ‘dispersion relations’ showing the dependence of $\omega _t$ on $k_p$ at $t=30$ for ${\bar{Q}}_H=2.5,3$, with all other parameters remaining unchanged from those used to generate Fig. 3. It is seen clearly that the dispersion curves are structurally similar to those shown in Fig. 1, associated with the linear stability of a film of uniform thickness, and exhibit low- and high-wavenumber cut-off ‘modes’, as well as a ‘most dangerous mode’ at an intermediate $k_p$ value. Increasing the value of ${\bar{Q}}_H$ leads to an increase in the wavenumbers associated with both the cut-off and most dangerous modes, as well as a rise in growth rate for the range of ‘unstable’ wavenumbers. This further highlights the fact that magnetic effects exert a destabilizing influence on the dynamics. In Fig. 6d, we also show the effect of the perturbation start time, $t_p$, on $\omega _t$ for ${\bar{Q}}_H=3.0$, and $t=25,30,35$. For the range of $t_p$ values shown, $\omega _t$ decreases with *t* (see also Fig. 6b) but appears to be relatively insensitive to $t_p$ for $t_p>10$. This result shows that the growth rates are not that sensitive to the perturbation start time even though the two-dimensional base state is evolving as the constant volume fluid spreads out and thins. Because the unperturbed flow is evolving with time there is a strong dependence in $\omega _t$ with time as shown in Fig. 6b. This result is in contrast with the constant flux configuration, where the unperturbed film reaches a steady state and the initial growth rate is a weak function of time.

**Fig. 7:**
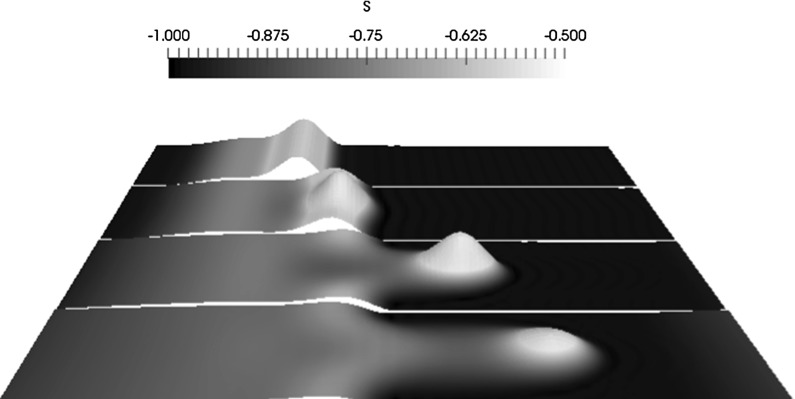
Surface plots of the spatiotemporal evovlution of the interface in the *x*–*y* plane at $t=10,36,100,$ and 200 (from *top* to *bottom*) for $k_p=1$ and ${\bar{Q}}_H=3.0$. The domain size is $2\pi k^{-1}_p\times 6 \pi$ and the rest of the parameters are unchanged from Fig. 3

In Fig. 7, we plot the spatiotemporal evolution of the interface associated with the application of a spanwise perturbation. At $t=t_p=10$, the drop has already spread down the incline, forming a capillary ridge at the downstream leading edge, and has just interacted with a spanwise, cosine perturbation of $k_p=1$ in its path. Due to the Maxwell pressure, the perturbation grows, as indicated by the results presented in Fig. 6, giving rise to a pronounced hump at the leading edge of the spreading drop. Since the magnetic field is oriented normal to the substrate, the peak of the hump is pulled in the wall-normal direction and a spike-like structure forms (see the surface plot associated with $t=100$ in Fig. 7). With the increasing time, the perturbed interface acquires the shape of a long finger, and the hump decreases in amplitude due to mass conservation. The large decrease in the amplitude of the finger over time is due to the spreading of a constant volume of fluid. In the constant-flux configuration, we expect to get a similar finger structure except that the height will probably not decrease as much over time.

**Fig. 8:**
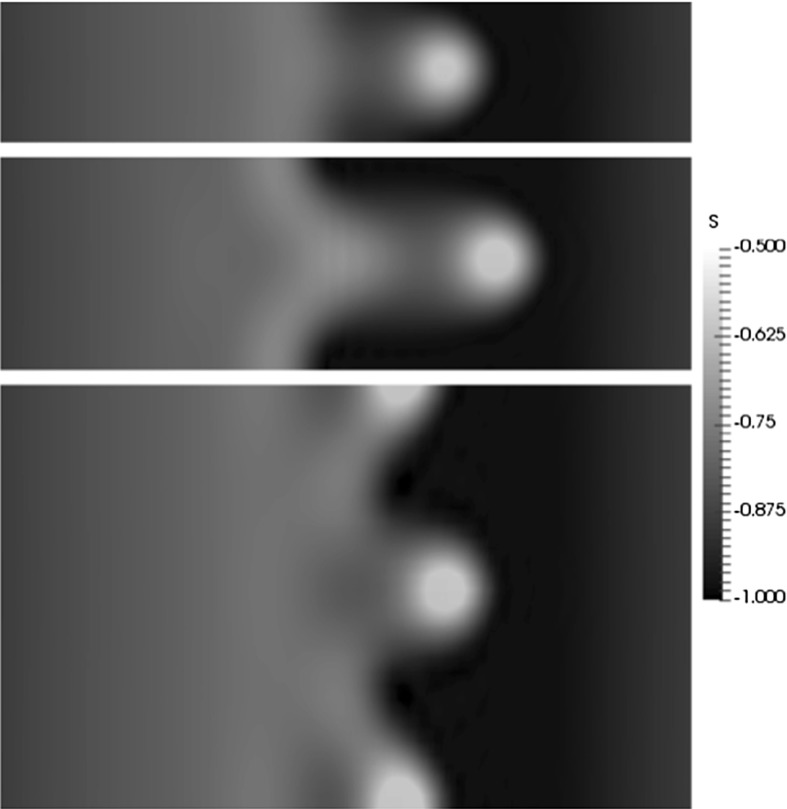
Surface plots (*top views*) of the interface in the *x*–*y* plane at a dimensionless time of $t=120$. Here $k_p=0.5$ (*bottom*), $k_p=1$ (*middle*), $k_p=1.5$ (*top*) and ${\bar{Q}}_H=3$. The domain size is $2\pi k^{-1}_p\times 6 \pi$, and the rest of the parameters are unchanged from Fig. 3

**Fig. 9:**
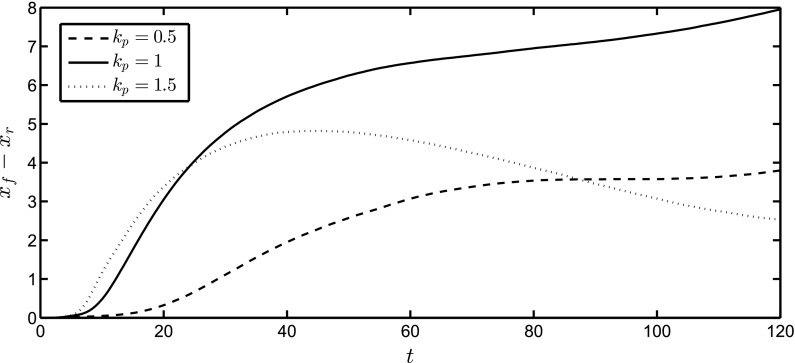
Temporal evolution of the finger length, the streamwise distance between the thickened finger hump and its root, $x_f-x_r$, for ${\bar{Q}}_H=3$, with the rest of the parameters remaining unchanged from Fig. 3

Surface plots (top views) of the interface for different spanwise perturbation wavenumbers are shown in Fig. 8. Here, $k_p=0.5,1,1.5, {\bar{Q}}_H=3$, and the rest of the parameters remain unchanged from Fig. 3. Since the domain size was chosen to fit one spatial period, only one complete finger is shown in each of the panels. The diameter of the head appears to have a similar size, in the *x*–*y* plane for all three $k_p$ values. In addition, the shoulder of the front is smaller for increasing values of $k_p$. The growth of the finger length with time, defined as the streamwise distance between the thickened finger hump and the finger root, is shown in Fig. 9 for $k_p=0.5,1$ and 1.5 and ${\bar{Q}}_H=3$. The fastest growing mode at early times occurs for $k_p=1.5$, which is consistent with the results presented in Fig. 6c. The finger growth at larger times is clearly much larger for $k_p=1$, and we see an acceleration phase for intermediate values of time, followed by a slower period that is likely due to the decrease in volume in the hump of the thickened finger.

#### Multiple spanwise modes

Next, we investigate the stability of the film to a large set of spanwise modes. In this way, the most dangerous mode will be naturally selected with the higher wavenumber content dampened by capillarity. In reality, the distribution of the perturbation will be non-uniform and environmentally dependent, but since the distribution is unknown, and we are interested in the self selection process, we use a uniform distribution. Also, we initiate the instabilities after a time $t_p$ following [22], since the time at which noise is imparted on the fluid is unknown.

**Fig. 10:**
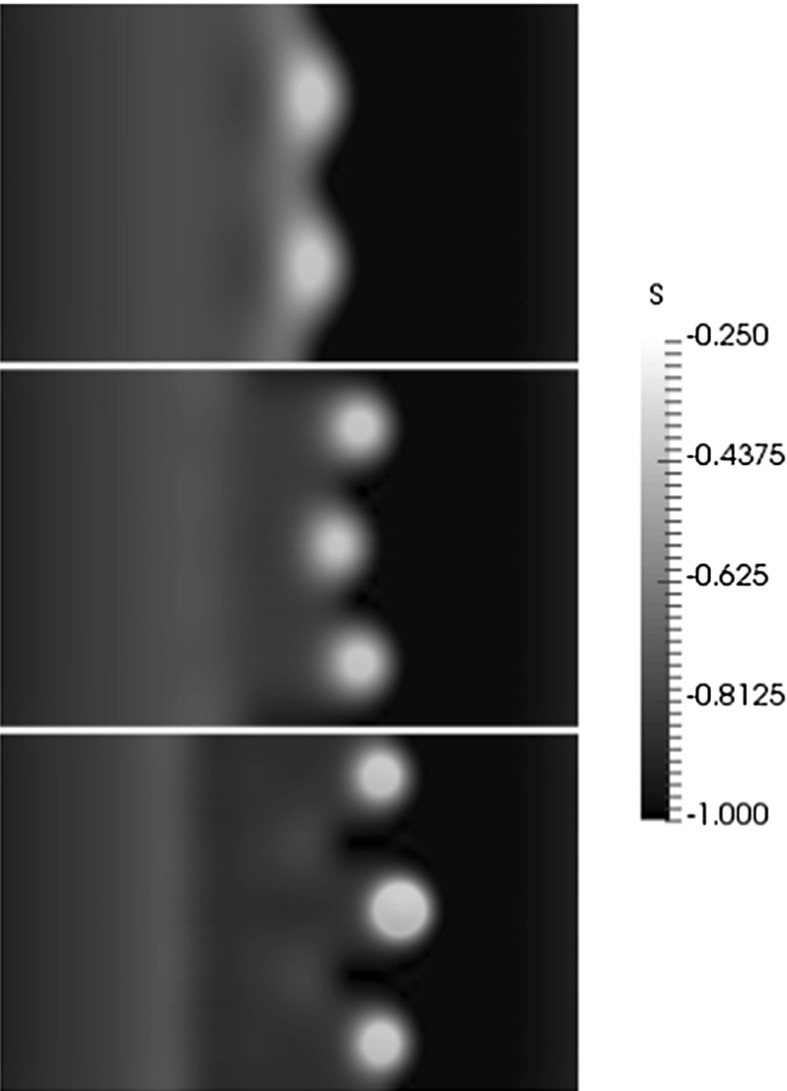
Surface plots (*top views*) of the interface in the *x*–*y* plane for multiple spanwise modes at a dimensionless time of $t=150$. The domain size is $4\pi \times 6 \pi$, and the parameter values are choosen to be ${\bar{Q}}_H=2.5$ (*top*), 3 (*middle*), and 3.5 (*bottom*), and the rest of the parameters are unchanged from Fig. 3

Surface plots of the interface are shown at $t=150$ in Fig. 10 for ${\bar{Q}}_H=2.5,3,3.5$. For small values of ${\bar{Q}}_H$, the interface profile looks similar to the non-magnetic case, consisting of a relatively smooth profile with thickened ridges at the leading edge. For large values of ${\bar{Q}}_H$, the drop velocity increases, and the tips of the fingers develop into spike-like structures. Also, for increasing ${\bar{Q}}_H$, the number of fingers increase indicating that the most dangerous wavelength decreases with a stronger magnetic field.

## Conclusions

In this paper, we have investigated the dynamics of a thin drop of a ferrofluid spreading down an inclined plane in the presence of a magnetic field oriented in a wall-normal direction. We have developed a model of the flow using a long-wave analysis for a viscous fluid accounting for Maxwell stresses. The magnetic field equations were solved for a relatively large magnetic permeability, and a magnetization that was assumed to be a nonlinear function of the magnetic field using a spectral technique. The stress singularity at the moving contact lines was relieved through the use of a thin precursor layer. For two-dimensional flow, we have shown that the spreading process is accompanied by the formation of a thickened ridge at the advancing contact line. With the increasing magnetic field strength, another hump is seen to develop upstream of the ridge. The spreading is observed to follow a power-law, which is consistent with gravity-driven spreading for the range of parameters investigated in the present study.

We have also examined the stability of the interface to the applied perturbations. We have derived a dispersion relation for the case of a flat ferrofluid film, which demonstrates the destabilizing effects of the magnetic field in the linear regime. We have also studied the stability of the two-dimensional spreading drops to spanwise perturbations of either single or multimodes, and shown that significant growth takes place leading to the formation of long fingers with spike-like ridges; the latter become particularly pronounced with increasing magnetic field strength. The results of this work will shed light on how magnetic fields can be used to manipulate fluids, which will be relevant to microfluidics and lab-on-a-chip applications.
